# Gleanings

**Published:** 1894-03

**Authors:** F. H. Lutze

**Affiliations:** Brooklyn, N. Y.


### GLEANINGS.

F. H. Lutze, M. D., Brooklyn, N. Y.

### Stool and Anus.

Stool but once a week, dark thick often with blood, has to strain, seizing thighs with hands, straightening himself forcibly till face is red, and head feels as if it would burst. Ind-met.

— hot, pouring away from anus like boiling water. Merc-sulph.

— — watery, daily. Calc-phos.

— hard, unnoticed and involuntary. Aloe.

— offensive and watery. Ars.

— — — slimy. Podoph.

Oozing from anus or rectum. Calc-c., Calc-phos., Carb-an., Ferr., Nitric-acid., Puls., Sep., Sil., Sulph., Thuja, Pæonia, Zinc.

Prolapsus, preceding a difficult stool. Ruta.

— and pain after stool. Rathan.

Urging constant, tormenting, unsuccessful, but increasing the pain and urging. Lachesis.

Diarrhœa in children, stool smells like rotten eggs. Æsclep-tub., Ars., Calc., Carlsbad., Cham., Fagopyr., Hepar, Psorin. (Puls.), Sulph., Sulph-ac., Wiesbaden.

Diarrhœa, chronic. Aloe, Guaranea, Petrol., Nahulus-alb. (Rattlesnake root) worse after breakfast, A. M., the stool feels hot.

Diarrhœa and vomiting, simultaneous. Iris-vers.

Stool a gob of mucus, in consistency like a jelly fish. Aloe.

— unnoticed, a well-formed, hard, and involuntary stool. Aloe.

Hæmorrhoids come down when urinating. Baryt-c.

Gurgling from stomach down through abdomen, causing involuntary stool from relaxed anus ; mouth and tongue dry and burning, constant desire for ice cold water in large quantities which is vomited, so soon as it becomes warm in the stomach. Phos.

Erosive pain during a rather loose stool, blood with stool, tenesmus after stool, frightful, continues some time after stool. (Nitr-ac.)—Violent burning at anus and rectum after a soft stool and great weakness; hæmorrhage from anus and rectum. Phos.

During stool darting pain from coccyx through spine as far as vertex, the head being drawn back by it; sharp scraping and burning at anus, frequently after stool, with burning desire to urinate without much urine being passed. Great relaxation in abdomen after a loose stool, giddy and near fainting after a second stool; violent tearing in rectum and genital organs; burning; continued cramp-like pushing around the rectum; cutting in anus and abdomen worse evenings; violent pain at anus, as if abdomen would be torn asunder, with cutting and movement of flatulence in abdomen, and a constant unsuccessful desire for stool; heat in hands and anxiety ameliorated by warm cloths; sensation in rectum in evening as if passage of fæces were prevented by something obstructing the rectum, the stool is not hard; rectum feels contracted during passage of fæces, which are not hard, an acrid, sore pain is felt in rectum, continuing several hours and extending to abdomen. Phos.

Sore pain in varices, when sitting or lying, for many days, with violent pressure and stitches in varices when rising. Phos.

Constipation, habitual; feeling of fullness in abdomen before stool, remaining after, as if all had not passed. Phos-ac.

— terrible straining to pass the stool; worse after drinking milk; it is covered with mucus; bloody discharge, slow and difficult discharge of even a soft stool; stool like sheep's dung, hard, knotty. Sepia.

— manual assistance is necessary on account of excessive straining to stool; obstinate constipation (in children and pregnancy), especially if Nux and Sulph. have failed. Sepia.

Perinæum, sense of weight in, and prolapse of anus during stool. Sepia.

Stool hot like boiled lead; ulcer of rectum; perinæal fistula. Thuja.

Obstruction from hardened stool; darting pains in inner parts; hæmorrhoids; want of perspiration. Verbascum-thaps.

### URINE AND URINARY ORGANS.

Bladder, pain in after urinating. Gamboge.

— inflammation of neck of, involving the muscular structure, either acute or chronic. Elaterium $1^x$, grs. ii, morning and evening.

Chill begins in neck of bladder at the end of urinating, and spreads over the entire body. Sarsap.

Cutting pains at close of micturition; knife-like pains at closure of the sphincter vesicæ at passage of last drop. Thuja.

Desire frequent and uncontrollable with bearing down; sense of fullness, discomfort, and pain; shuddering over whole body with desire to urinate; urethra feels like a hard rubber tube, and is sore, sensitive, and tender. Hypericum.

— — with urging to urinate, and pressure on bladder, all relieved by riding on horseback; bearing-down pains. Lycopod.

— — to urinate worse at night, the quantity is small, offensive; can urinate only when standing; sensation as if urethra had turned to bone. Hypericum.

Gonorrhœa or gleet, for last persistent drop. Natr-mur.

Involuntary urination, when under excitement or emotion. Cina.

Pregnancy, cystic symptoms during. Puls.

Urging so sudden he cannot make the necessary preparations quick enough; yet if compelled by surroundings, he can overcome the desire for a time. Thuja.

— to urinate on arising from a sitting position. Carbo-veg.

Air gurgling from urethra, when urinating. Sarsap.

Child pulling and elongating penis. Merc-v.

Standing, can urinate only when. Sarsap.

Enuresis nocturna. Ars., Graph., Caust., Lycopod., Natr-c., Natr-m., Petrol., Puls., Senega, Psor., Gels., Bell., Ferr., Phos.

Pellicle on top of urine, variegated. Iod., Phos.

— — — — — greasy. Paris-quad., Sulph.

— or cuticle simply. Psor., Sep.

— — — gray. Lycopod.

Pellicle or cuticle slimy. Petrol.

White pellicle or film on urine, which smells strong. Kali-bi.

Urine excoriates genitals, inside of thighs, and flow is painful to parts it passes over. Sulph.

Paralysis of bladder, predisposition to from over-distention. Apis, Sulph. (Puls., Hell., Aletris, Caul.)

Urine, absence of, in the new-born. (Ars.) Scream constantly. Apis.

Sediment as fine as flour, seldom gritty; depositing on vessel in red rings easy to wash off. Colchic-aut.

Urinate, desire to, with shuddering. Hypericum.

Urine frequent; urgent desire but no flow till after waiting some time; desire worse mornings; enuresis during first sleep. Sepia.

Disuria, distress at neck of bladder; burning pain; sleepless, with reflex bladder trouble from uterine irritation; prolapse or flexion. Senecio.

Urinate, must frequently; during paroxysms of pain must rise and urinate, most frequently from five to nine P. M. Thuja.

### Male Sexual Organs.

Chancre, phagedenic, gangrenous; anguish and restlessness. Ars.

— — quiet and composed. Merc-cor.

— — very angry looking. Cinnabar.

Crawling, creeping sensation in the scrotum. Clematis.

Impotence. Argent., Cobalt., Digit., Staph., Ustilago, Zinc.

Itching, scrotum, child pulling at scrotum. Staph.

Pulling and elongating penis. Merc-v.

Gonorrhœa, or gleet, for persistent last drop. Natr-mur.

Prostatic fluid, discharge of. Alum., Ammon-c., Ars., Calc., Cann-ind., Carb-v., Caust., China, Con., Ignat., Kali-bi., Nux-m., Phos., Staph.

Respiration difficult during coition. Arundo-m.

— — toward the end of coition. Kali-bi., Kali-c., Staph.

— — after coition. Arundo-m., Asafœtida, Staphys.

— — on attempting coition. Ambra-gris.

Sitting, when, a numb feeling creeps up from knees to scrotum and penis. Baryta-c.

Walks slowly with legs stretched apart, for the urethra hurts him, when walking as usual, worse from motion; (in gonorrhœa) Cann-sat.

Pains sharp and cutting, through scrotum to root of penis. Conium.

Prepuce inflamed as if distended with water or air. Merc-cor.

Syphilis, pinkish-red swellings of the tibia, nodes with unbearable pain. Kali-iod.

— ulcers of roof of mouth. Aurum.

— single small tumor of roof of mouth, somewhat involving bone. Mangan-acet.

— many small tumors of roof of mouth, with suppuration; the tumors are discolored and the bone deeply involved in suppuration. Asafœt.

— bluish nodes on tibia. Mangan-acet.

— — — — — small and numerous, affecting periosteum. Asafœt.

Sycosis, condylomata of vulva, perinæum, anus, and internal genitals, deeply fissured, seedy, like cauliflower, on cervix with thick greenish leucorrhœa, the warts often ooze a gelatinous fluid, ulcers appear often like chancroids on vulva and near parts; ulceration if from warts, fissures, and furrows about anus or perinæum. Thuja.

— ulcerations of the vulva, the ulcers have a deep hard base, and red, inflamed areola; a tenacious discharge accumulates around the genitals. Kali-bi.

— greenish leucorrhœa, moist fissure at anus, bones ache where they are devoid of muscular tissue, as on tibia. Nitri-acid.

— filiform condylomata, crumbling of teeth, eczematous eruption with yellow scabs. Staphys.

— condylomata itch and burn. Sabin.

— — like cockscomb. Euphras.

— — fanshaped. Cinnabar.

— moist eruption around the genitals. Sarsaparil.

— — — — — and membranous shreds around anus. Petrol.

### Female Sexual Organs.

Abortion, precursory symptoms. Arn., Bell., Bry., Cann-s., Cham., China, Coccul., Croc., Hydrast., Hyos., Ipec., Kali-c., Lycopod., Nux-v., Plat., Plumb., Puls., Rhus, Ruta, Sil., Sulph., Zinc.

Before menses, sadness. Amm-c., Bell., Berb., Caust., Con., Cyclam., Ignat., Lac-c., Lac-def., Lycopod., Natr-m., Nitr-ac., Phos., Puls., Sep., Stan., Xanthæ.

Cutting, shooting, and squeezing in left ovary. Thuja.

Delay or too scanty first menses. Calc-c., Puls., Sep., Sulph.

Flushes of heat, followed by coldness. Lachesis.

— — — — — perspiration. Sulph.

— — — — — mental depression. Cimicifuga.

Leucorrhœa, in children, infantile leucorrhœa. Calc-c., Cann-s., Iod., Merc-cor., Puls., Teucrium.

Menses suppressed from anæmia, chronic diseases of the uterus or ovaries, and subjective symptoms due to it. Polygonum hydropiperoides (also for diseases of mucous surfaces, nervous system, fibrous tissues, and urinary apparatus, as loss of expulsive power, suppression, strangury, paralysis of bladder from over-distention. Polygon-hydro.)

Menses only during the night. Bovista.

— — — — day. Causticum.

— increasing at night. Amm-c., Zinc.

— none at night. Causticum.

— only mornings. Sepia.

— — evenings. Coffea.

— less afternoons. Magnes-c.

— increasing —. Sulph.

— in the daytime, mostly when walking. Caustic., Puls.

— suppressed with albuminuria. Helon.

— in the morning only. Sepia.

— — — — more profuse. Bovista.

— cease in the afternoon. Magnes-c.

— increase in the afternoon. Puls., Sulph.

— in the evening only. Coff., Phelland.

Menses during the day only. Caust., Ham.

— when walking only, not during rest. Lil-tig.

— only when lying, cease when walking. Magnes-c.

— cease when lying. Cact., Caust., Lil-tig.

— at night only. Bovista, Brom.

— — — increase. Amm-c., Magnes-c., Zinc. (Amm-m.)

— only at night, or only in the morning or profuse mornings, scanty day and night. Bovista.

— not copious, but last longer than usual and only mornings, similar to Sepia. Carbo-an.

— morning and evening. Phelland.

Metrorrhagia, large black lumps, worse from motion, with violent pains in groins, fear of death, despair, bright red face; fever. Coffea.

Ovarian irritation, in nervous, excitable women, during temporary absence of husband. Staphis.

Ovarian region, pain in left, shoots down in upper parts of thighs and up the left side, strikes across stomach, and makes her sick, with bearing down. Bell.

Puerperal fever. Sulph.

Sensation of swelling of uterus; it feels as if dropsical. Saracen.

Uterine irritation, sleeplessness and bladder trouble. Senecio-aur.

— hæmorrhage, in a girl only ten years old. Cina.

Vagina too small during labor, hardly admits the index finger. Ars.

